# Synergism therapeutic and immunoregulatory effects of Albendazole + rAd-mIL-28B against Echinococcosis in experiment-infected mice with protoscoleces

**DOI:** 10.1371/journal.pntd.0009927

**Published:** 2021-11-24

**Authors:** Yan Zhang, Jianghua Wang, Qingxia Yang, Zhi Li, Xiaoying Xu, Chong Chen, Zongjie Hou, Qi He, Li Sheng, Xingming Ma, Yanping Luo

**Affiliations:** 1 Department of Immunology, School of Basic Medical Sciences, Lanzhou University, Lanzhou, China; 2 Center for Drug Safety Evaluation and Research, School of Pharmacy, Zhejiang University, Hangzhou, China; 3 Department of Immunology, School of Medicine, Northwest Minzu University, Lanzhou, China; 4 Gansu Provincial Key Laboratory of Evidence Based Medicine and Clinical Translation & Lanzhou Center for Tuberculosis Research, School of Basic Medical Sciences, Lanzhou University, Lanzhou, China; University of Passo Fundo: Universidade de Passo Fundo, BRAZIL

## Abstract

The metacestode stage of *Echinococcus granulosus* can cause cystic echinococcosis (CE), which still widely occurs around the world. Since the early 1970s, benzimidazoles have been shown to inhibit the growth of cysts and used to treat CE. However, benzimidazoles are still ineffective in 20%-40% of cases. In order to explore the new agents against CE, we have investigated the therapeutic effect of the recombinant adenoviral vector expressing mouse IL-28B (rAd-mIL-28B) on protoscoleces*-*infected mice. In our study, we successfully established the model mice which infected with protoscoleces intraperitoneally. At 18 weeks post-infection, the mice received rAd-mIL-28B (1×10^7^ PFU) weekly by intramuscular injection for 6 weeks. Compared with the untreated control (13.1 ± 2.2 g), there was a significant reduction in cysts wet weight in rAd-mIL-28B group (8.3 ± 3.5 g) (*P* < 0.05), especially in Albendazole (ABZ) + rAd-mIL-28B group (5.8 ± 1.4 g) (*P* < 0.01). We also observed the severe damage of the germinal layer and the laminated layer of cysts after treatment. rAd-mIL-28B group showed a prominent increase in the level of Th1 type cytokines (such as IFN-γ, IL-2 and TNF-α). Meanwhile, the frequency of Foxp3^+^ T cells was decreased in the rAd-mIL-28B group (4.83 ± 0.81%) and ABZ + rAd-mIL-28B group (4.60 ± 0.51%), comparing with the untreated group (8.13 ± 2.60%) (*P* < 0.05). In addition, compared with the untreated control (122.14 ± 81.09 pg/ml), the level of IFN-γ significantly increased in peritoneal fluid in the rAd-mIL-28B group (628.87 ± 467.16 pg/ml) (*P* < 0.05) and ABZ + rAd-mIL-28B group (999.76 ± 587.60 pg/ml) (*P* < 0.001). Taken together, it suggested that ABZ + IL-28B may be a potential therapeutic agent against CE.

## Author summary

Echinococcosis is a chronic zoonotic parasitic disease, which is caused by the larval stage of *Echinococcus granulosus* (*E*. *granulosus*) and *Echinococcus multiocularis* (*E*. *multiocularis*). This disease is still widely prevalent in the world and seriously endangers human health and life, causing heavy burdens and economic losses to agriculture and animal husbandry. China is also one of the high incidence areas of the disease. At present, the preferred treatment is surgical excision of the parasitic mass, but patients with multiple cysts appear in multiple organs have to receive drug therapy. The approved chemotherapeutic drugs in clinic, such as albendazole (ABZ) and mebendazole, often do not work expectedly with a high rate of recurrence. Therefore, it is urgent to develop the new anti-echinococcal drug. IL-28B is an important member of type-III IFNs, which is equipped with the capacity of anti-viral activity and anti-tumor. In our previous study, we found that IL-28B could inhibit the proliferation of cervical cancer cells via down-regulating Treg cells in mice. This study mainly studied the therapeutic effect of rAd-mIL-28B on *E*. *granulosus*-infected mice, and the results showed that rAd-mIL-28B could relieve the parasitic burden and inhibit Treg cells meanwhile improve the Th1 and Th17 immune responses. It may contribute to another choice for the anti-echinococcal treatment.

## Introduction

*Echinococcus granulosus* sensu lato metacestodes is the causative agent of cystic echinococcosis (CE), as known as hydatid disease [1]. The life cycle of *Echinococcus granulosus (E. granulosus)* involved two mammalian hosts. The eggs of *E. granulosus* were ingested by the intermediate hosts (sheep and cattle), developing into a hydatid cyst, which containing protoscoleces (PSCs). The definitive hosts (such as dogs) got infected by swallowing the tainted tissue from intermediate host and PSCs can develop into adult tapeworms in small intestine, residing for a long time [2]. It reported that the echinococcal cysts can survive in human body for 20–25 years, even as long as 50 years [3], which caused the long-term damage and burden in patients.

Mostly, CE is located in the liver of patients and parasitic tissues are characterized by unilocular and fluid-filled cysts [3]. The cyst is composed of cyst wall, cystic fluid, PSCs and brood capsules. The cyst wall mainly consists of an outer acellular laminated layer and an inner layer of germinal cells, and the later can produce vesicles into the cyst lumen [4].

CE also is one of the neglected parasitic diseases in the world, its effective treatment is not yet available [2]. Since the early 1970s, benzimidazoles have been shown to inhibit cysts growth and used to treat CE [5]. Albendazole (ABZ) and mebendazole, which belong to the category of benzimidazoles, are the only drugs approved for use in humans [6]. But such chemotherapeutic drugs often do not work expectedly. After decades of clinic practice, it has been found that benzimidazoles are ineffective in 20%-40% of cases [7]. Even it reported that only 30% of patients were cured after the benzimidazoles treatment, which accompanied with instable efficacy and great individual differences. Besides, a long-term treatment causes the side-effects [6], including hepatotoxicity, alopecia, gastrointestinal disturbances and leukopenia [8]. Therefore, it is urgent to develop a new anti-hydatid drug agent with high-efficiency and few side effects.

Immune response plays a crucial part in patients with various kinds of parasites, in general, Th1 immune response was regarded as the protective immunity, while the growth of parasite is corresponding to Th2 response [9]. It reported that in the mouse cysticercosis model [10], the host shows up a protective Th1 response accompanied by high level of IFN-γ and eases the burden of parasite during the early infection; whereas, the long-term infection induces Th2 response that favors parasite survival, characterized by high levels of IL-4 and IL-6. Moreover, in the acute phase of Schistosomiasis [11], parasite antigens contribute to Th1 response along with increased levels of IFN-γ and TNF-α, which benefits the early granulomas. In contrast, a long-lasting Th2 response induces the anti-inflammatory and the high levels of IL-4, IL-5, and IL-13 while IFN-γ is decreased. Consistent with the above cases, after the *E*. *granulosus* infection, the cysts induce the Th1 response with the high levels of IFN-γ and IL-2 in early period; while the Th2 response accompanied with the high level of IL-10 that caused the cysts survival after weeks [12]. One possible reason is that direct inflammatory effects are hard to eliminate the parasite, because of the worms being large [13]. Meanwhile, the infection can balance out the Th1-mediated pathologies and promote Th2-bias, which regulate the Th2 response via stimulation of Treg cells populations [13]. The anti-hydatid immunity has been recognized step by step, but it has not been fully elucidated because of the complexity [14]. Due to the importance of immune response in the outcome of echinococcosis, a number of biological agents, such as BCG, IL-12 and IFN-γ, had been revealed certain efficacy against hydatid in mice [15]. But it had not been implemented in the clinic because of the unstable efficacy and side effects.

IL-28B is a member of type-III IFNs. Type-III IFNs also named IFN-λ which consists of IFN-λ1 (IL-29), IFN-λ2 (IL-28A) and IFN-λ3 (IL-28B) [16]. IL-28B was revealed the capacity of anti-viral activity and anti-tumor, in accordance with type-I IFNs due to the similar signal transduction cascade [17]. Our previous data indicated that IL-28B inhibits the growth of cancer via down-regulating Treg cells [18]; and rAd-mIL-28B can reduce the population of Treg cells in tuberculosis subunit vaccine-immunized mice [19]. These findings indicated that IL-28B equipped with immunomodulatory activity.

In this study, we illustrated the therapeutic effect and immune mechanism of the recombinant adenoviral vector expressing mouse IL-28B (rAd-mIL-28B) on *E*. *granulosus* infection model mice. After 6 weeks treatment, we detected the weight of the cysts, morphologic changes and ultramarine structure of cysts, T-cell subsets and Th1, Th17-type cytokines to evaluate the potential effects on CE.

## 2 Materials and methods

### 2.1 Ethics statement

The in vivo experimental were carried out according to the protocols (number. 2015–03–002) approved by the Institutional Animal Caring and Using Committee of Lanzhou University.

### 2.2 Animals

**Fig 1:**
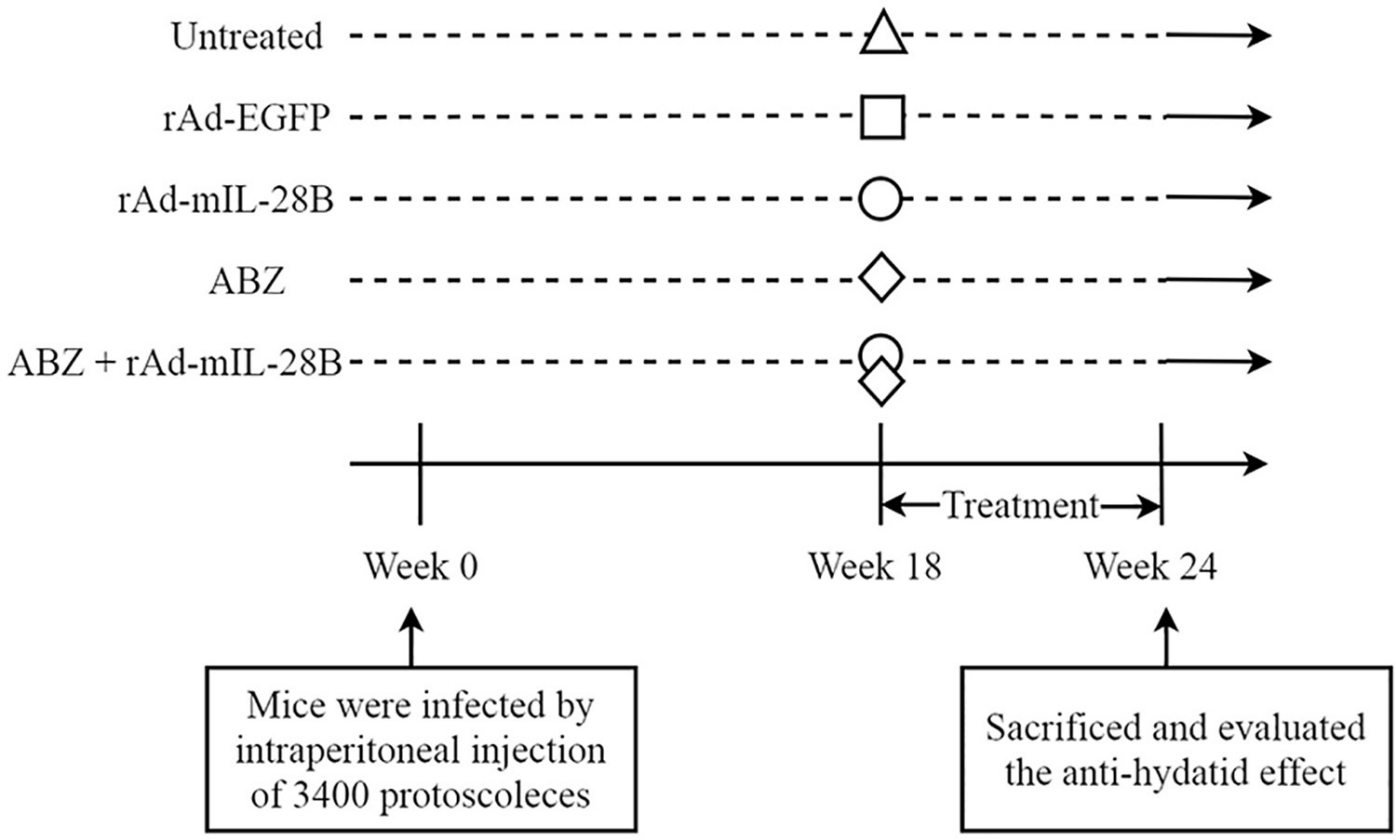
Experimental procedures. Mice were infected by intraperitoneal injection of 3400 protoscoleces at week 0 and treated by the rAd-mIL-28B or ABZ alone and ABZ + rAd-mIL-28B at week 18. The untreated mice were just given PBS at week 18. rAd-EGFP was used as a control for rAd-mIL-28B. Six weeks after the last administration, all mice were sacrificed to evaluate the anti-hydatid effect by the weight of the cysts, morphologic changes and ultramarine structure of cysts, T-cell subsets and Th1, Th17-type cytokines.

In this work, those mice were used to study the anti-hydatid effect of IL-28B in vivo (Fig 1). Thirty special pathogen-free (SPF) BALB/c mice (18–22 g, female) were purchased from Experimental Animal Center of Lanzhou University and received intraperitoneal inoculations with *E*. *granulosus*. Mice were maintained in the SPF Grade Trial Animal Center (Lanzhou University, Lanzhou, China). The PSCs were originally obtained from a sheep naturally infected with *E*. *granulosus* in Xining, Qinghai province, China. Mice received free access to food and water throughout the study. Mice were infected by intraperitoneal injection of 3400 PSCs in 200 μL of phosphate buffered saline (PBS). All experiments were carried out according to the protocols (number. 2015–03–002) approved by the Institutional Animal Caring and Using Committee of Lanzhou University.

### 2.3 Groups and treatment

After the 18 weeks of infection, the thirty infected mice were randomly divided into five groups, including the untreated group, the rAd-EGFP group (the recombined adenoviral vector encoding enhanced green fluorescent protein, as control of rAd-mIL-28B), the rAd-mIL-28B group, the ABZ group and the ABZ + rAd-mIL-28B group. Six mice were enrolled in each group (n = 6).

The treatment as followed: the untreated group was only given PBS daily for intervention; the rAd-EGFP group received rAd-EGFP 100 μL (1×10^8^ PFU/ml) weekly by intramuscular injection; the rAd-mIL-28B group was injected rAd-mIL-28B 100 μL (1×10^8^ PFU/ml) intramuscular weekly; the ABZ group was given ABZ (100 mg/kg/day) daily by intragastric administration; and the ABZ + rAd-mIL-28B group were received ABZ (100 mg/kg/day) by intragastric administration daily and rAd-mIL-28B 100 μL (1×10^8^ PFU/ml) by intramuscular injection weekly.

The general appearance of the mice was observed daily during the entire experiment, including mental condition, food and water intake, urine and stool. Upon 6 weeks of treatment, the animals were euthanized by cervical dislocation under narcosis with 0.3% pentobarbital sodium (10 mL/kg) by intraperitoneal injection.

### 2.4 The weight of the cysts was measured

The cysts were dissected out carefully and immediately weighed, the inhibition rate of cysts were calculated based on the following formula: Inhibition rate of cysts (%) = untreated wet weight (g)—treated wet weight (g) / untreated wet weight (g) × 100%.

### 2.5 Hematoxylin and eosin (H&E) staining

The cysts were fixed in 4% paraformaldehyde overnight at room temperature and used to stained with H&E, as previous described [20]. In briefly, the fixed cysts were washed with PBS and dehydrated with alcohol gradient (50%, 75%, 95% and 100% ethanol). After paraffin-embedded and cut into slices, the tissues were stained with H&E. Then, the morphological characteristics of cysts were observed under light microscopy (Olympus, Tokyo, Japan).

### 2.6 Scanning electron micrograph (SEM)

Cysts from mice were fixed in 2.5% gluteraldehyde for 24 h at 4 °C and processed for SEM [21]. Then the cysts were fixed in 2% osmic acid (OsO_4_) for 1.5 h at 4 °C and washed with PBS three times, the cysts were dehydrated with alcohol gradient (50%, 70%, 80%, 90% and 100% ethanol) for 10 min each. The cysts were placed in the refrigerated drying chamber overnight. After freeze-drying, they were fixed on the aluminum stubs and coated with a layer of gold. The coated samples were observed under scanning electron microscope (JSM-5600LV, Japan) with accelerating voltage of 10kV and 20 kV.

### 2.7 Flow cytometry analysis

The spleen of each mouse was removed, and minced in Ficoll-Hypaque Solution, which was performed as described previously [22]. After centrifugation for 40 min at 800×g, spleen cells sediment were washed with 10 mL of RPMI 1640. The lymphocytes were resuspended in flow cytometry stainning buffer and the concentration was adjusted to 5×10^7^/mL. After twice washing, lymphocytes were processed with ice-cold Fixation/Permeabilization Diluent for 12h at 4°C. The samples were resuspended with 1×Permeabilization Buffer after centrifugating for 5 min at 400×g. Next, the cells were incubated with the APC—labeled anti-mouse Foxp3 (1μL/1×10^6^ cells) was performed for 30 min at 4°C, followed by washing. Finally, the cells were resuspened with Wash buffer and analyzed by an ACEA NovoCyte flow cytometer (ACEA, America).

### 2.8 Cytokine quantification by array

After anesthesia by using 0.3% pentobarbital sodium, the blood samples were obtained by eyeball removal. The serum was separated from blood by the centrifugation at 3000×g for 30 min and used to test cytokines. This array is designed to detect 17 cytokines, including IL-6, IL-23, IL-17, IL-12p70, IL-17F, IFN-γ, IL-5, IL-10, IL-21, IL-4, TGF-β1, IL-2, IL-22, TNF-α, MIP-3α, IL-28 and IL-13. The cytokines were examined using the Quantibody Mouse TH17 Array 1 Kit (RayBiotech, Inc, Guangzhou, China) according to the manufacturer’s instructions. The data were digitized and analyzed with the microarray analysis software (GenePix, ScanArray Express, ArrayVision, MicroVigene, etc.).

### 2.9 Enzyme-Linked Immunosorbent Assay (ELISA)

The peritoneal fluid was obtained as described [23]. Briefly, after anesthesia by using 0.3% pentobarbital sodium, the mice were intraperitoneally injected with 1mL PBS and the abdominal was opened immediately. The lavage was collected and stored at -80°C until use. Then, the peritoneal fluid and serum were used to test the concentration of cytokines: IL-10 and IFN-γ. ELISA was performed according to the instructions of the kits producers. An ELISA dedicated instrument was used at 450 nm for the measurement of optical densities, all determinations were performed in duplicate [21].

### 2.10 Statistical analysis

Statistical analysis was done with IBM SPSS Statistics 22. One-way analysis of variance (ANOVA) was used. Data were shown as mean ± standard deviation (SD) of the mean. *P*-value < 0.05 was considered statistically significant.

## 3 Results

### 3.1 ABZ + rAd-mIL-28B treatment reduced the parasite burden in mice

Mice were presented with quick action, glossy hair and normal stool and urine. All the animals survived to the end of the experiment, and there was no accidental death.

**Fig 2:**
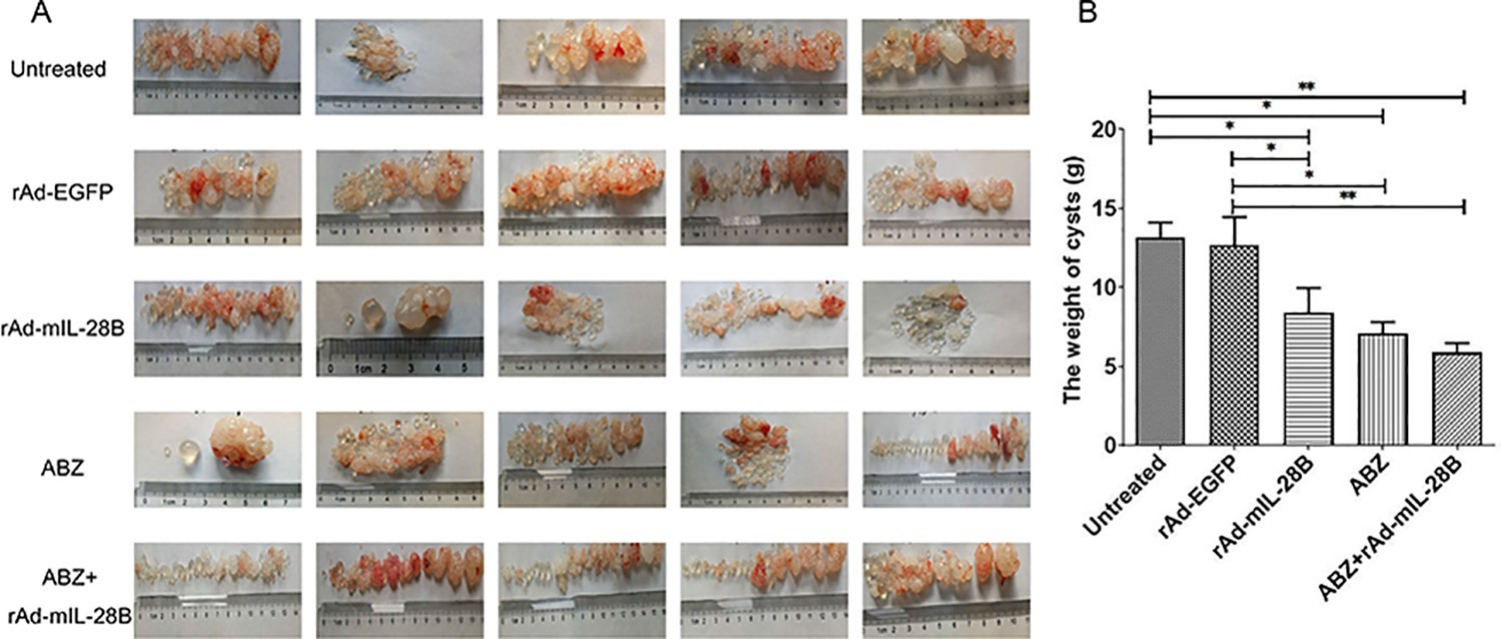
ABZ + rAd-mIL-28B have shown therapeutical effects against *E*. *granulosus* in vivo. The BALB/c mice were infected with protoscoleces intraperitoneally. After 18 weeks of infection, the infected mice received rAd-mIL-28B treatment for 6 weeks. The mice were euthanized and parasitic tissues were dissected out (n = 5). (A) The appearance of hydatid cysts. (B) The wet weight of hydatid cysts. * represented *P* < 0.05, ** represented *P* < 0.01 and *** represented *P* < 0.001. Graph shows the means ± SD.

At the sixth week after the last administration, the abdominal cavity was opened and the parasitic tissue was carefully removed after euthanasia. A numerous of unilocular fluid-filled cysts with different sizes were found in each experimental mouse. Meanwhile, the cysts in the mice treatment with rAd-mIL-28B showed less blood vessels compared with rAd-EGFP-treated mice. This phenomenon was even more pronounced in the ABZ + rAd-mIL-28B group, including the less blood vessels and number of cysts (Fig 2A).

To investigate the therapeutic effect of rAd-mIL-28B, the cysts were weighted and inhibition rate of cysts was calculated. The cysts wet weight was significant difference in mice treated with either rAd-mIL-28B (8.3 ± 3.5 g) or ABZ (7.1 ± 1.7 g) (*P* < 0.05), compared to untreated group (13.1 ± 2.2 g) (shown in Fig 2B). Furthermore, the result showed a prominent reduction of the cysts wet weight in ABZ + rAd-mIL-28B group (5.8 ± 1.4 g) (*P* < 0.01) (Table 1).

**Table 1:** The wet weight and inhibition rate of hydatid cysts in rAd-mIL-28B treated mice.

Group	Weight (g)	Inhibition rate of cysts	*P*-value
Untreated	13.1 ± 2.2	0%	/
rAd-EGFP	12.6 ± 4.0	3.8%	0.752
rAd-mIL-28B	8.3 ± 3.5	36.6%	0.007
ABZ	7.1 ± 1.7	45.8%	0.001
ABZ + rAd-mIL-28B	5.8 ± 1.4	55.7%	0.000

### 3.2 ABZ + rAd-mIL-28B treatment damaged the germinal layer (GL) and the laminated layer (LL) of cysts

**Fig 3:**
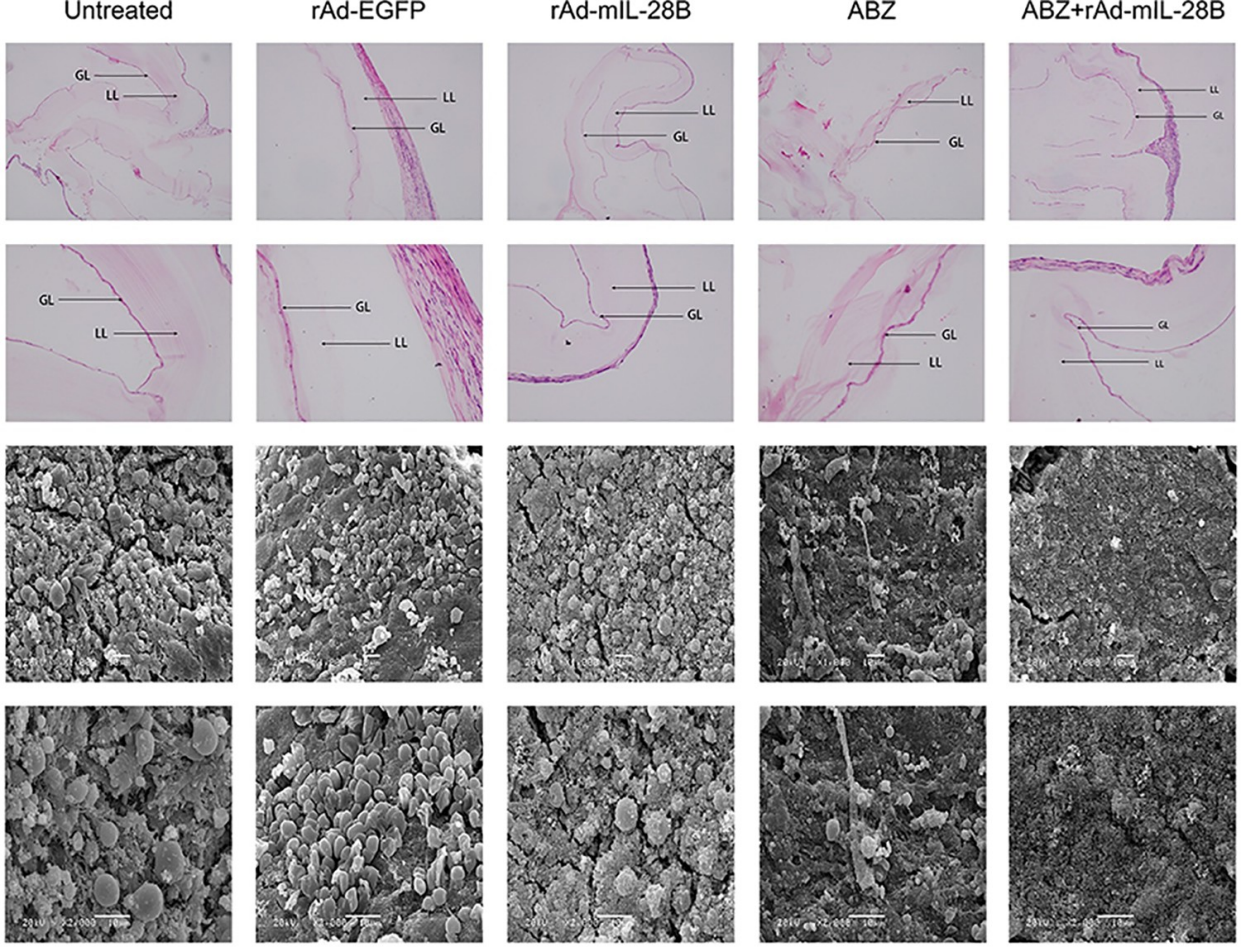
ABZ + rAd-mIL-28B induced the morphological changes of cysts in *E*. *granulosus*-infected mice. The *E*. *granulosus-*infected mice were euthanatized after 6 weeks of treatment with rAd-mIL-28B and the cysts were removed carefully. The morphological changes of cysts were observed under light microscopy after stained with H&E (the upper two lines) and the ultrastructures were observed under scanning electron microscope (the bottom two lines).

To investigate the morphologic changes, the cysts were observed by microscope after H&E staining. The untreated cysts showed an intact germinal layer, texture clear laminated layer, and distinct border between the germinal layer and the outer acellular laminated layer. In the rAd-mIL-28B group and the ABZ + rAd-mIL-28B group, the germinal layer both showed apparent damage, including lacked the typical structure and the reduction in cell number. Besides, there were many lymphocytes in the outer adventitia, it indicted the rAd-mIL-28B could induce the recruitment of lymphocytes to an anti-parasite effect during the treatment (Fig 3).

The therapeutic effect can be further confirmed on the ultrastructural level by SEM. All cysts from untreated mice showed an intact and typical structure in ultrastructure: the cells arranged in neat rows with regular morphology in the germinal layer, and the laminated layer attached closely with the germinal layer, accompanying the integrity of cellular structure (Fig 3). However, the cysts isolated from rAd-mIL-28B group and the ABZ + rAd-mIL-28B group were markedly altered: germinal layer was lost the integrity of cellular shape and detached from the laminated layer partly. In addition, the obvious fractures were observed in rAd-mIL-28B group. Meanwhile, ABZ + rAd-mIL-28B treatment induced the more distinct damages.

### 3.3 ABZ + rAd-mIL-28B treatment decreased the frequency of Foxp3^+^ T cells in spleen

**Fig 4:**
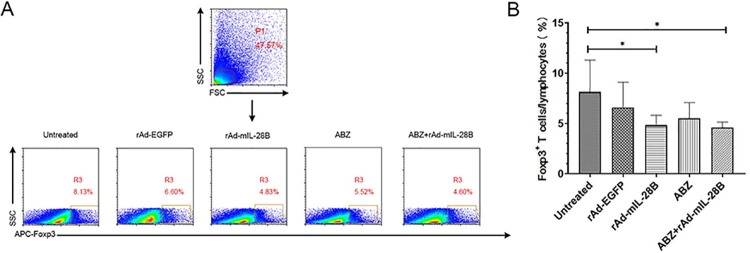
The percentage of Foxp3^+^ T cells in spleen. The *E*. *granulosus*-infected mice were euthanatized after 6 weeks of treatment with ABZ + rAd-mIL-28B. Then the lymphocytes of spleen were isolated and the Foxp3^+^ T cells were detected by FCM (n≥3). (A) Mice of per group were analyzed and the representative of three independent experiments is shown. (B) Data collected from each group were expressed as means ± SD. * represented *P* < 0.05, ** represented *P* < 0.01 and *** represented *P* < 0.001.

To study the influence of immune function of IL-28B, we examined the functional activity of Foxp3^+^ T cells in spleen. The results showed that the frequency of Foxp3^+^ T cells was decreased in the rAd-mIL-28B group (4.83 ± 0.81%) comparing with the untreated group (8.13 ± 2.60%) (*P* < 0.05). Also, the ABZ + rAd-mIL-28B group (4.60 ± 0.51%) decreased significantly when compared the untreated group (*P* < 0.05) (Fig 4).

### 3.4 rAd-mIL-28B treatment increased the levels of Th1, Th17-type cytokines

**Fig 5:**
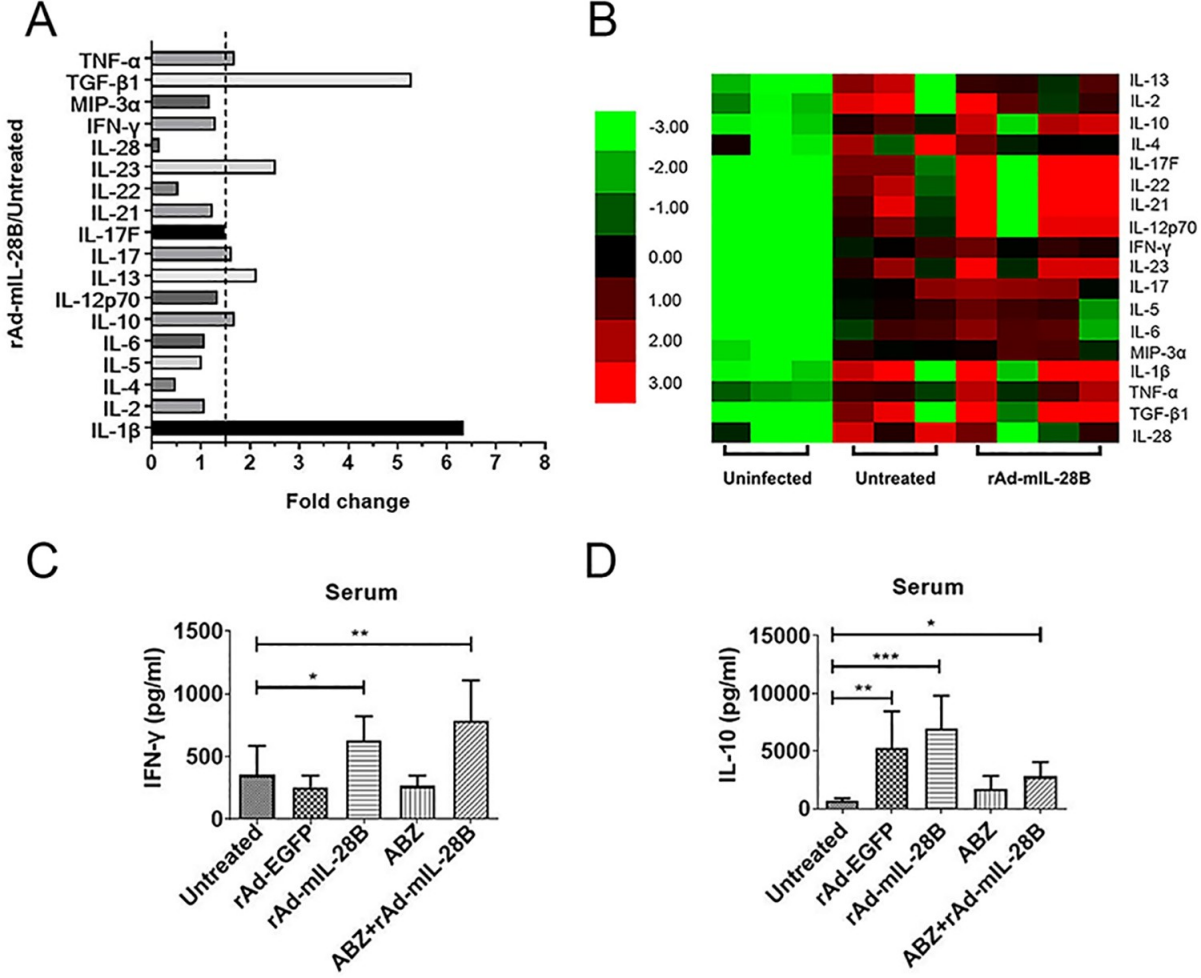
The levels of Th1, Th2 and Th17-type cytokines. The infected mice were received various treatment for 6 weeks and the serum was separated (n≥3). The serum was collected to detect cytokines with ELISA and cytokine quantification by array. (A) Th1, Th2 and Th17-type cytokines of rAd-mIL-28B group / untreated group ratio. (B) The heat map of cytokine expression. (C) Concentration of serum cytokine IFN-γ of mice in each group. (D) Concentration of serum cytokine IL-10 of mice in each group. * represented *P* < 0.05, ** represented *P* < 0.01 and *** represented *P* < 0.001. Graph shows the means ± SD.

In the present experiment, the result clearly showed that the level of IFN-γ, Th1-type cytokines, was significantly increased in the rAd-mIL-28B group. And the levels of Th17-type cytokine (IL-23, IL-17, IL-17F) were prominently increased (*P* < 0.05), comparing with untreated group (Fig 5A). Meanwhile, the other cytokines, such as TNF-β1 and IL-1β, present more than twofold increased expression. It also revealed that the Th2-type cytokine IL-4 was decreased in the rAd-mIL-28B group (Fig 5B).

### 3.5 ABZ + rAd-mIL-28B treatment increased the level of IFN-γ in peritoneal fluid of *E*. *granulosus-*infected mice

**Fig 6:**
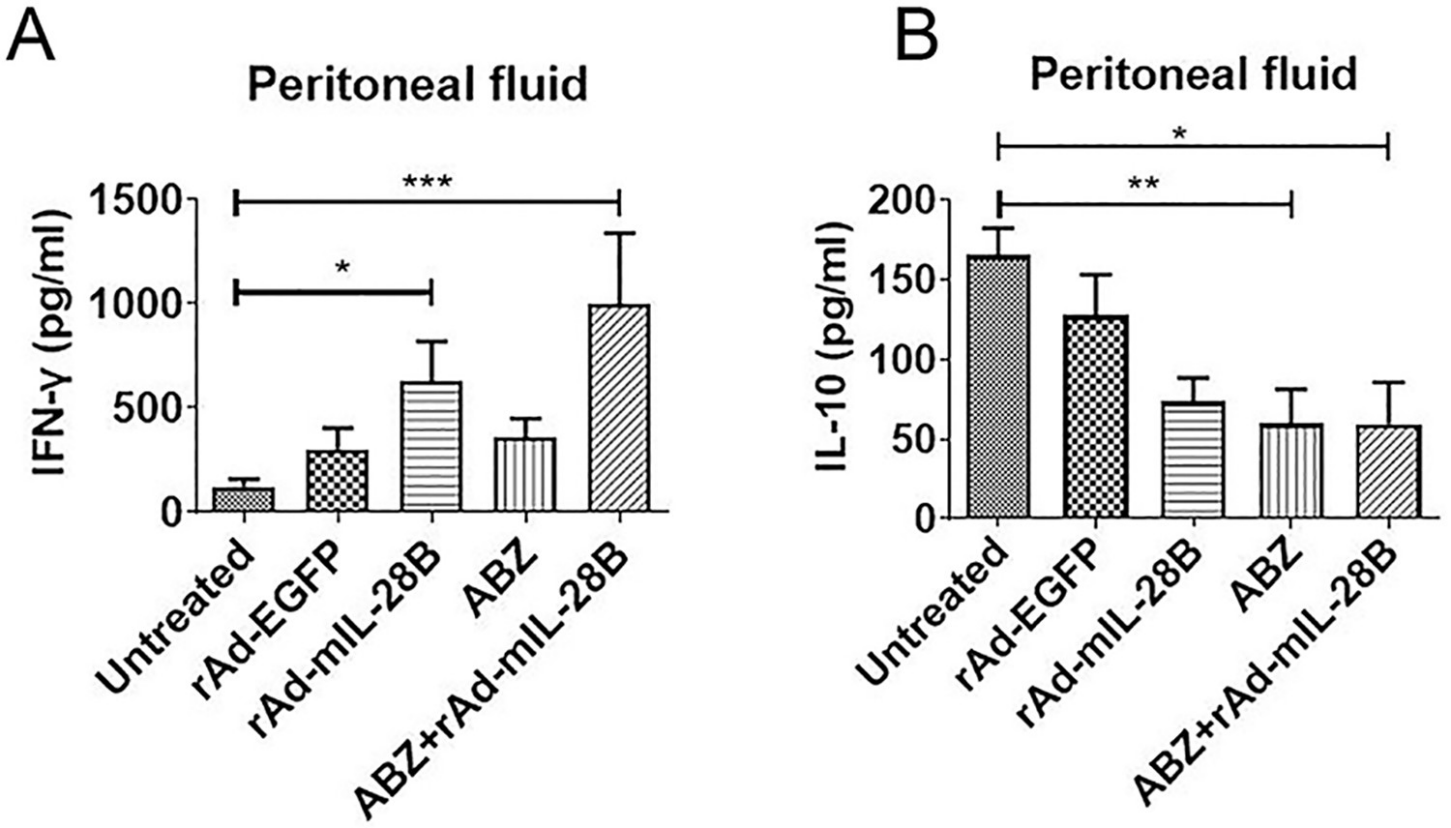
The concentration of cytokine IFN-γ and IL-10 in peritoneal fluid of *E*. *granulosus-*infected mice. At 18 weeks post-infection, the *E*. *granulosus-*infected mice were euthanatized after 6 weeks of treatment with ABZ + rAd-mIL-28B. The mice were intraperitoneally injected with 1mL PBS and the abdominal was opened immediately. The lavage was collected and used for ELISA (n≥3). (A) Concentration of cytokine IFN-γ. (B) Concentration of cytokine IL-10. * represented *P* < 0.05, ** represented *P* < 0.01 and *** represented *P* < 0.001. Graph shows the means ± SD.

In this test, the *E*. *granulosus-*infected mice were euthanatized after 6 weeks of treatment with rAd-mIL-28B, and the peritoneal fluid were collected through injecting PBS in abdominal cavity, then the concentration of cytokines: IFN-γ and IL-10 were test by ELISA. The result indicated that the concentration of IFN-γ increased remarkably (*P* < 0.05) in rAd-mIL-28B group compared with untreated group, especially in ABZ + rAd-mIL-28B group (*P* < 0.001). In contrast, the concentration of IL-10 decreased significantly (*P* < 0.05) in ABZ + rAd-mIL-28B group. There was a decreased trend of IL-10 in rAd-mIL-28B group, but it was not statistically significant (Fig 6).

## Discussion

Cystic echinococcosis (CE) is a zoonotic helminthiasis caused by the larval stages of the *E*. *granulosus*. So far, it still regarded as a major economic and public health concern around the world [24]. Up to now, the first choice treatment agent is surgery, which has been associated with the high rate of recurrence. As for benzimidazoles, there are many studies have proven that the severe side effects and drug resistance for the patients [25]. Therefore, it is urgent to find a new and useful agent. In this study, we demonstrated that rAd-mIL-28B can decrease the frequency of Treg and inhibit of Th2 cell activation in *E*. *granulosus* infection model, suggesting that IL-28B may be a useful therapeutic agent for CE.

Previous studies have shown that IL-28B exhibits an immunomodulatory activity. For example, it reported that [26] IL-28B is an important regulator of the Th1/Th2 balance by increasing Th1-type cytokines (IFN-γ) and decreasing Th2-type cytokines (IL-4, IL-5 and IL-13) during influenza vaccination. Morrow [27] found that IL-28B led to increased cellular immune responses when used as an adjuvant in DNA vaccination. Further, IL-28B reduces Treg cell-mediated immunosuppression by the adaptive immune system [28]. Majumder [29] proposed that IL-28B alone or in combination with IL-27 can decrease lung nodules number and increase the activity of CD8^+^ cells in lung cancer bearing mice. In summary, these literatures suggested the IL-28B may be a regulator of the immune response. Our previous studies have shown that IL-28B can inhibit the growth of cervical cancer in U14 tumor-bearing mice by down-regulating Treg cells [18]. In addition, we also found that the rAd-mIL-28B down-regulated Treg cells in tuberculosis subunit vaccine-immunized mice [19]. In this study, the *E*. *granulosus*-infected mice were euthanatized after 6 weeks of treatment with rAd-mIL-28B, the reduction of parasitic tissues and the damage of cysts were observed. At the same time, the level of Th1-type cytokines (IFN-γ) in serum and peritoneal fluid was significantly increased, which accordance with the previous studies.

Treg cells are a subset of CD4^+^ T cells [30] which play an important role in inhibiting immune response, especially cellular immunity [31]. It worked through humoral and cellular immunity to inhibit the immune response [32]: consumption of IL-2 stimulate the suppressive activity of Treg cells [33]; cytotoxic T lymphocyte antigen 4 (CTLA-4) expressed in Treg cells affected the ability of antigen-presenting cells (APC) to activate other T cells [34]; adenosine are characterized by suppressing T cell proliferation, which generating from CD39 and CD73 on Treg cells [35]; Treg cells also can product the immune inhibitory cytokines and molecules [32]. In addition, Treg cells suppress anti-tumor immune response, which are harmful to the body. Studies have proven that tumor tissues were infiltrated with a large number of Treg cells that is thought to be associated with a poor prognosis [36]. Therefore, reducing Treg cells numbers seems more likely to be a targeted mechanism of CE. In this present study, we found that the Treg cells number decreased significantly in the rAd-mIL-28B group and ABZ + rAd-mIL-28B group compare to the untreated group (*P* < 0.05), and the severe damage of the germinal layer could be seen. This may indicate that the reduced Treg cells are helpful for anti-hydatid disease which verified the literature we talked about. Th17 cells are a CD4^+^ T cell subset and usually beneficial to host defense by secreting IL-17, IL-22, and IL-23 [37]. Recent studies have shown that the polarization of the immune response to Th1/Th17 is a key factor in anti-hydatid immunity, whereas Treg cells are contribute to survival of echinococcosis [38]. Thus, enhancing the Th17 response is an important anti-hydatid factor. Generally, the differentiation of naive CD4^+^ T cells into Th17 cells depends on TGF-β together with IL-6 and IL-21; whereas, the TGF-β alone drives differentiation into Treg cells [39]. Hence, the balance of Th17 and Treg cells is necessary for immune homeostasis; in other words, the decreased frequency of Treg cells accompanying with the elevated Th17 immune response. It reported that patients with hepatic echinococcosis had an imbalance of Th17/Treg, especially in the recurrence of CE, which may be involved in the establishment of the disease and persist into the advanced stages of the disease [40]. There are many literatures have shown that decreased Treg cells are conducive to anti-tumor immune response [32]. In the present study, we noted that the frequency of Foxp3^+^ T cell in spleen was decreased, meanwhile the levels of Th17-type cytokines were increased after the rAd-mIL-28B treatment, which showed a beneficial immune characteristic against hydatid disease.

In recent years, consensus has gradually emerged that *E*. *granulosus* cysts induce the secretion of Th1-type cytokines (IFN-γ and IL-2) in an early period, which can shift toward a Th2-type profile (IL-4, IL-5 and IL-6) after few weeks in mice model [12]. Clinical studies have shown that when the effective anti-parasite treatment leads to the infertile of cysts, it mainly associated with the Th1 immune response. On the contrary, a failed anti-parasite treatment will contribute to the Th2 response, and accompanied with the high level of IL-10, which caused the cysts survival [41,42]. In mice model, IFN-γ and IL-12 enhanced Th1 immune response and decreased the cyst load index, whereas IL-4 boost Th2 immune response and increased the cyst load index [43]. After the *E*. *granulosus* infection, the parasite led to an increase in the number of Treg cells with the high levels of IL-10 and TGF-β [44], which disrupting host immune function and causing immune escape.

In conclusion, ABZ + rAd-mIL-28B reduced the parasitical burden markedly compared to rAd-mIL-28B alone in *E*. *granulosus-*infected mice and inhibited Treg cells meanwhile improved the Th1 and Th17 immune responses. It suggests that ABZ + IL-28B had the potential to be used as a treatment of CE in the future. However, the limitations of analysis are obvious in our experiments, such as failure to detect the transformation of immune cells in thymus and design drug toxicological evaluation. We will continue to solve these problems in future studies.
